# Exploring How Rheumatic Fever Is Portrayed on TikTok: A Descriptive Content Analysis

**DOI:** 10.3390/ijerph22050686

**Published:** 2025-04-26

**Authors:** Siobhan Tu’akoi, Malakai Ofanoa, Samuela Ofanoa, Maryann Heather, Hinamaha Lutui, Felicity Goodyear-Smith

**Affiliations:** 1 Pacific Health Section, School of Population Health, University of Auckland, Auckland 1023, New Zealand; 2 Etu Pasifika, Auckland 2104, New Zealand; 3 Southpoint Family Doctors, Auckland 2104, New Zealand; 4 Department of General Practice and Primary Health Care, University of Auckland, Auckland 1023, New Zealand

**Keywords:** rheumatic fever, TikTok, content analysis, social media, video

## Abstract

TikTok is a popular social media platform offering educational opportunities for health issues such as rheumatic fever, which primarily affects 4–19-year-olds globally. This content analysis aimed to explore the type of rheumatic fever content available and popular on TikTok and the role that rheumatic fever representation may play in shaping public understanding and attitudes. The top 100 TikTok video posts under the hashtag #rheumaticfever were examined. Descriptive statistics were used to summarize video metrics and deductive thematic analysis enabled the coding of video content. The majority of TikTok users creating rheumatic fever content were patients or family members of people suffering from rheumatic fever (42%), followed by health professionals (30%). Forty-three percent of videos had negative connotations and personal stories were the most commonly coded type of video (42%). In terms of rheumatic fever content, symptoms (*n* = 59), medications/treatment (*n* = 37) and disease pathogenesis (*n* = 36) were the most common themes. Misinformation was identified in 3% of videos. This study provides a unique insight into who is making rheumatic fever-related content on TikTok and the primarily negative framing of narratives people are exposed to. There are opportunities for future health promotion strategies to focus on the gaps identified in this study, including information on where to seek health services, primordial prevention and stories of recovery.

## 1. Introduction

With almost three billion users worldwide, social media platforms have emerged as popular forums for sharing content and conversations online [1]. A study of 1296 young people (13–18 years) in the United Kingdom found that 53% used social media to look for health content and 46% reported changing a health behaviour as a result of seeing something on social media [2]. More recently, governments and health organisations harnessed such platforms during the COVID-19 pandemic to disseminate health messages on a large scale and to inform public behaviour [3]. However, alongside the positive outcomes, there is also potential for the sharing of misinformation or increased stigma if health content is communicated poorly [3]. Social media platforms provide a unique opportunity to educate young people, dispel misinformation and contribute to health-promoting behaviours. TikTok is a social media application founded in 2017 which has become one of the fastest-growing platforms globally, particularly for young people. It is estimated that currently there are more than 800 million current users, with 64% under the age of 29 years [4]. TikTok is video-based and has a main “For You” page providing recommendations based on a user’s algorithm. Compared to other social media platforms which primarily focus on connecting people who know each other, the TikTok algorithm is based on audience preferences, thus allowing unfamiliar users to disseminate content and information widely [5]. As TikTok becomes increasingly popular, it is important to understand the types of information and narratives being shared and how this can impact public perceptions of different health issues.

Rheumatic fever is a disease that occurs due to an untreated Group A streptococcal infection, typically affecting children and adolescents aged 4–19 years across sub-Saharan Africa, the Middle East, South East Asia, and Indigenous and Pacific populations in Australia and New Zealand [6,7]. A review outlined that the most common clinical presentations of acute rheumatic fever include joint pain, choreiform movements, and acute fever, tiredness and breathlessness from cardiac failure [8]. Repeated damage to heart valves from rheumatic fever can lead to the development of rheumatic heart disease—the most common heart disease globally for people under the age of 25 years [7]. Although risk factors are complex and strongly linked to socio-economic determinants [6], most cases can be treated successfully with timely antibiotics if identified early. However, gaps in public understanding persist globally regarding rheumatic fever, prevention and related treatment [9,10,11]. In efforts to improve outcomes for young people, a scoping review of interventions to date in New Zealand for example, found they ranged from school-based screening programmes to national mass media and awareness campaigns [12]. However, past media campaigns which intended to promote awareness, may have contributed to further stigmatization and inequity for the most affected groups [13]. It is thus important to scrutinize how this health issue is presented in the media in order to understand the narratives communities are exposed to and to better tailor interventions.

Content analysis is one approach to analyzing the meanings, context, and consequences of communication and media [14]. More than just a simplistic description of data, content analyses involve analysing the presence, meaning and relationship of concepts to produce new insights, identify misconceptions in the media and form practical guides to action [14,15]. Although such analyses can review diverse sources of information, visual and auditive content have been less commonly analysed compared to written content [15]. With the broad popularity of social media and video formats such as TikTok among young people, text-based media analyses are no longer sufficient to meet modern needs [16]. A range of health issues have been investigated using content analyses methods [17,18,19,20], highlighting gaps and areas of opportunity for health promotion strategies. For example, a content analysis focused on how official public health accounts on TikTok communicated COVID-19 information during the pandemic [21]. The most prevalent theme coded across videos was personal precaution measures taken by individuals to prevent COVID-19 and the dance videos were found to be the most popular. The study concluded that public health agencies should be cognizant of TikTok for health communication and develop audience-centred risk communication [21]. Despite many examples of content analyses on infectious and chronic diseases, little is known about streptococcus infections or rheumatic fever content, despite this disease remaining a significant health issue for many children and adolescents globally. This cross-sectional study aimed to explore the type of rheumatic fever content available on TikTok, and how this might influence public understanding and attitudes.

## 2. Materials and Methods

### 2.1. Data Collection

The hashtag #rheumaticfever was searched on a TikTok phone application. A new account was created to limit the potential effects of previous searches, viewing history and user engagement, all of which impact a user’s algorithm [18,22]. A sample size of 100 videos was determined prior to data collection based on other content analysis studies and due to the increasing number of irrelevant videos being identified towards the end of data collection. The default sorting mode under the hashtag #rheumaticfever was used to reflect the behaviour of health consumers who typically view the top search results when seeking online information [23]. All videos were retrieved on 14 May 2024 to limit the impact of changes in the algorithm. In total, the #rheumaticfever hashtag had 253 TikTok video posts at the time of data collection. Data collection occurred until 100 videos were retrieved under the following inclusion criteria: videos clearly referencing rheumatic fever, in the English language and posted up until the retrieval date. To gain a sample of 100 relevant videos, 178 were reviewed with 78 excluded for reasons including being irrelevant, in languages other than English, or with information that was duplicated or irretrievable. Types of data collected included the video, caption, hashtags, date posted, creator username, type of creator, number of account followers, country of origin, length of video, and number of likes, comments, bookmarks, and shares.

### 2.2. Analysis

Quantitative analysis using video popularity metrics and descriptive statistics were conducted on IBM SPSS Statistics 28 (International Business Machines Corporation, New York, NY, USA) to determine average numbers of views, likes and comments. A coding framework was then developed (Appendix A) to deductively code the textual, visual and auditive content of video posts. Framework categories were sourced from literature and adapted after a preliminary review of the first 10 videos. The coding framework explored information on who is providing or presenting the content, the type of video, the primary purpose of the video, tone, overall connotations, and the key rheumatic fever themes. Multiple codes were permissible where appropriate and all coding considered the caption, video content and any on-screen text. Independent assessment of video posts was conducted by two reviewers (S.T. and S.O.) to determine video relevance and ensure consistency of coding. A high level of intercoder reliability was achieved using Cohen’s kappa, whereby all values were above 0.75 indicating strong agreement. Discrepancies were discussed to reach consensus, with a third reviewer (F.G.S.) available if required.

## 3. Results

Most rheumatic fever content originated from New Zealand-based creators (38%), followed by 31% from the United States, 11% from the Philippines, 2% from Kenya and 1% each for Egypt, the Democratic Republic of Congo and Canada (country of origin was unidentifiable for 15% of the sample). Table 1 outlines key descriptive statistics for the top 100 videos, which totalled 748,143 views, 25,590 likes and 2137 comments. The final sample comprised 53 unique creator accounts, with follower numbers ranging from seven to 821,100. The length of videos ranged from six seconds to seven minutes, with the average TikTok lasting a minute and 10 s. Engagement in rheumatic fever TikToks averaged 256 likes, 22 comments and 12 bookmarks and shares per video post. These variables were positively skewed, with most videos concentrated towards lower values and only a few outliers exhibiting exceptionally high values.

Table 2 outlines the key characteristics of the video content that was coded in this analysis.

### 3.1. Who Is Presenting Content?

The most common types of presenters coded in this analysis were rheumatic fever patients or close family members of patients (42.2%). As a result, the primary purpose of the videos were personal stories (42.4%), with patients discussing their own symptoms and experiences of treatment pathways. For example, one TikTok by a United States based creator presented a montage of video clips showcasing her rheumatic fever symptoms and the time in hospital recovering. Floating text captions on the video outlined, “The day that my life changed forever. I relapsed with rheumatic fever. It targeted my brain, heart and joints. I lost the ability to use my legs. I went numb on the left side of my body and suffered from memory, hearing and sight loss”.

Health professionals or those in medical training, such as paediatricians, cardiologists and registered nurses were the second most common presenter of rheumatic fever TikTok content (30.4%). Despite this, videos by health professionals had a higher mean number of views (*n* = 12,973) and followers (*n* = 55,198) compared to videos by patients (*n* = 6160 and *n* = 8382 respectively). Health professional-led videos typically focused on education and awareness of rheumatic fever, giving health advice and discussing their own clinical experiences of the disease. For example, one TikTok presented a paediatrician explaining the potential severity of a streptococcal throat infection to viewers, “The bacteria that causes strep throat actually goes to other parts of the body…it can go into the heart, and it can cause permanent damage in the heart valves. That is what we are trying to avoid with antibiotics… to try and prevent rheumatic heart disease”. Less common presenters of rheumatic fever TikTok content included general community members (such as school students, church groups and a psychic) a former professional athlete and media podcasters.

### 3.2. Rheumatic Fever Content

**Figure 1:**
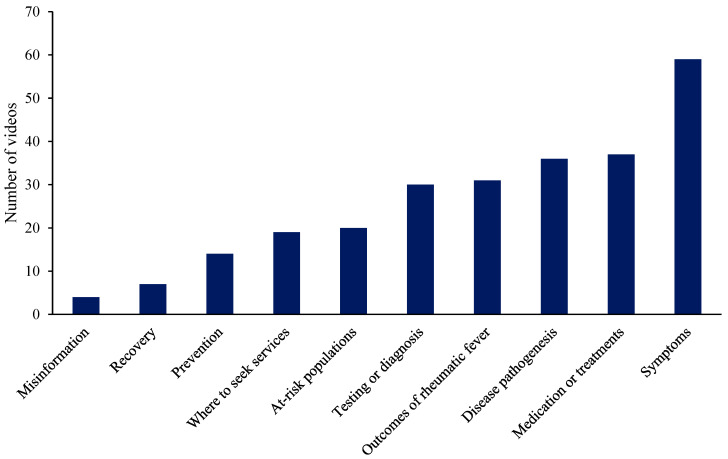
Frequency of rheumatic fever content themes in TikTok videos. Note: Seven videos were coded as themeless or mere mention of rheumatic fever and were not included in the figure.

Figure 1 presents the rheumatic fever content themes coded from the TikTok videos in this analysis. References to symptoms of rheumatic fever and streptococcus infections (*n* = 59), medications and treatments (*n* = 37), and disease pathogenesis (*n* = 36) were the most prevalent topics discussed.

Symptoms of rheumatic fever and streptococcus infections was the most common theme coded (*n* = 59), with videos referencing sore throats, fevers and unusual jerky movements of the body. One creator with rheumatic fever discussed her symptoms and asked whether viewers had similar experiences, expressing, “I feel like I’m being weighed down with every step or movement. I just need to sleep or rest. Anybody else sleep like 10 h last night and still feel like they could go right back to bed? I’m exhausted”. Medications and treatments were the second most prevalent theme, with TikTok videos ranging from patients documenting themselves receiving monthly intramuscular injections to community members talking about antibiotics and home remedies. TikTok videos by health professionals that focused on treatments and medications often emphasized the importance of adherence, with one general practitioner outlining, “Just one simple injection or 10 days of antibiotics and you’re done. But you’ve got to make sure that you do the full 10 days to prevent yourself from developing rheumatic fever”.

Less common topics covered in rheumatic fever TikToks included discussions on at-risk populations (*n* = 20), where to seek health services (*n* = 19), primordial prevention (*n* = 14) and the recovery journey (*n* = 7). While not widespread throughout the collected data, three cases involving misinformation were identified, including direct misinformation (*n* = 1 layperson), and reference to or correcting misinformation (*n* = 2 health professionals). One layperson creator described a friend who claimed to have cured her rheumatic fever-related heart damage by opting for folk medicine rather than surgery, “She needed to obtain a live chicken…and then they would make chicken soup. She drank all the chicken soup… the heart had completely mended, there was no problem”. Another video under this code showed a cardiologist stitching a response to a different TikTok creator, who had claimed to have cured her daughter’s strep throat by getting her to drink potato juice. In his response video, the cardiologist described the danger of misinformation and how strep throat can potentially be life-threatening if left untreated by antibiotics, “I see a lot of dumb [things] on TikTok every day, but this video is the unique combination of not only being stupid but also having downright dangerous information that can be harmful for children… don’t listen to TikTok for medical advice”.

## 4. Discussion

Rheumatic fever affects children and adolescents globally; however, little has been explored on how it is framed and presented in different media. This study is the first descriptive content analysis focused on rheumatic fever, and specifically its portrayal on TikTok. In general, presenters of content tended to be people affected by the disease, sharing their stories visually or verbally. A study analysing TikTok chronic pain content reported similar findings, with over 80% of the video sample being created by patients and for the purpose of expressing their own experiences and raising awareness, rather than to share solutions or remedies [24]. More broadly, young people report that sharing their lived experiences on TikTok helps them to cope with feelings of fear, frustration and uncertainty, such as during the COVID-19 pandemic [25]. The implications of having patients share personal stories on social media can be positive, including feeling connected and part of a community. Health organisations and practitioners wanting to disseminate messages on rheumatic fever and other adolescent health issues could prioritise and support the sharing of real-life stories in the context of accurate health advice to develop engagement and build a sense of community on these platforms.

While the dominance of patients as creators of TikTok health-related content provides important understandings of lived experiences, it may also foster the spread of misinformation, if creators have limited understanding or access to health advice. In this analysis, 3% of videos were deemed as referencing misinformation, with advice including using bleach, drinking potato juice and eating chicken soup. Comparatively, a content analysis of health videos from #EduTok found that 14% of videos were framed as debunking misinformation and these videos gained greater interactions than those which did not discuss misinformation [26]. A United States study surveyed over 1000 young women regarding their perceptions of health information on TikTok and found that although 98% believed misinformation was prevalent on the application, only 55% of respondents believed that they had encountered it [27]. The authors suggested that this may be due to a “third-person effect” whereby respondents perceived media messages as having more influence on other people, and potentially naïve to the effects on themselves [27]. Literature shows that low levels of issue knowledge, scientific knowledge and cognitive ability are key factors that can impact susceptibility to misinformation on social media, alongside emotional states, worldviews and psychological motivations [28]. This presents an opportunity for researchers and health practitioners to be informed of the public discourse around different health issues and use media platforms to both counteract false information and improve health literacy.

Symptoms of rheumatic fever and streptococcal infections were the most commonly referenced theme, with creators sharing their experiences of fevers, joint pain and fatigue. As a result, content was often coded as having negative connotations, highlighting themes of pain, suffering and regrets of not engaging with health services earlier. Many TikTok videos placed the onus on parents to be proactive and take sick children to the doctor, with one video even comparing it to abuse and neglect. Negative representations of health issues are common in the media, with a study on palliative care content finding that print media coverage heavily focused on euthanasia (74%) and prolonged suffering, and less so on how palliative care can improve patient quality of life (17%) [29]. Deficit framing can contribute to misconceptions and negative views in the public, which in turn can influence health behaviours [29,30]. Health organisations have an opportunity to balance the narrative with more strengths-based messaging and be intentional with health promotion approaches, so as to avoid unintentional fearmongering or stigmatization [31]. Rheumatic fever topics that could be promoted further and which were identified as gaps in this analysis include primordial prevention strategies, stories of recovery and advice on where to seek help, support and services. The implications of not having a strong presence of these topics on media platforms could result in a lack of awareness and understanding of prevention and which health services can be accessed. Although videos that overly highlight symptoms and pain are portraying the lived experience of individuals, it may unintentionally create negative narratives of the disease in the absence of content focused on areas of help and support. As predominant users of social media, it is important that adolescents and young people are equipped with high-quality health information to empower agency over seeking preventative and support services, rather than inciting fear.

### Strengths and Limitations

To the best of our knowledge, this analysis is the first to review media content related to rheumatic fever and thus provides an important contribution to the body of knowledge on both rheumatic fever and the potential of social media platforms to drive public health awareness. Using a hashtag search of English language videos aligned with other content analyses and ensured that videos were more likely to be relevant to the topic of rheumatic fever. However, a limitation is that videos in other languages or without this hashtag may be missed from the sample even if they may be relevant. Another limitation of this study is that a large proportion of videos were generated from a small number of TikTok accounts. For example, one account run by a health provider based in New Zealand had 31 of their TikTok videos in the 100-video sample. Despite this, we felt it was important to include all relevant top 100 videos to mimic what an adolescent user searching for rheumatic fever information might see. There is inherent selection bias in focusing only on TikTok, as the platform is primarily for entertainment purposes rather than health education and there is no way to verify truthfulness of information being presented, such as if users are in fact qualified health professionals as they appear to be. It also limits the representativeness of the data for other social media applications, such as Instagram, X (formerly Twitter) or Facebook. Future research could look across different media platforms to further investigate the rheumatic fever content being disseminated by health organisations specifically and how these narratives might influence public perception. Further qualitative research with adolescents could explore how they directly perceive rheumatic fever messages and what types of approaches they believe are engaging for young people.

## 5. Conclusions

Overall, social media provides a key opportunity to engage in public narratives of how health issues are framed. For rheumatic fever and other health issues that primarily impact young people, social media applications such as TikTok may provide an important forum to engage and spread awareness. In particular, there are opportunities for rheumatic fever interventions to emphasize the gaps highlighted in this study, namely information on where to seek health services, primordial prevention and stories of recovery. However, this should be carried out carefully to avoid further stigmatization and stereotyping and, further, should prioritise content made by youths, for youths. Researchers, health professionals and health promoters have an opportunity to incorporate the use of social media platforms for health-related messaging in order to best meet the needs of adolescents in spaces where they are comfortable.

## Figures and Tables

**Table 1:** Descriptive statistics of top 100 rheumatic fever-related TikTok posts.

	Mean	Median	Range	Total
Video length	1:10	0:49	0:06–7:03	1:54:55
Views	7481	746	22–223,500	748,143
Likes	256	19	0–16,200	25,590
Comments	22	0	0–1383	2137
Bookmarks	12	1	0–538	1156
Shares	12	0	0–839	1220
Creator followers	21,804	756	7–821,100	2,180,363

**Table 2:** Characteristics of TikTok videos.

Characteristics		*n*	%
Presenter	Patient with rheumatic fever or close family member	43	42.2
	Health professionals (registered and training)	31	30.4
	Community member	18	17.6
	Public figure (e.g., celebrity, professional athletes)	1	1.0
	Media personnel	1	1.0
	Unclear	8	7.8
Video type	Oral speech or singing	47	45.6
	Visual documentary	21	20.4
	Pictures or pictorial slideshow	12	11.7
	Animated infographics or illustrations	7	6.8
	TikTok trends and memes	12	11.7
	Acting/role play	4	3.9
Audio	Direct speaking or singing	54	54
	Voice over	16	16
	Music or sounds	27	27
	No audio	3	3
Primary purpose of video	Health advice	26	23.9
	Personal stories	46	42.2
	Medical education	15	13.8
	For entertainment	22	20.2
Overall connotation	Positive/positive leaning	27	27
	Neutral	30	30
	Negative/negative leaning	43	43
Tone	Serious	29	20.6
	Scary	8	5.7
	Sad	16	11.3
	Light-hearted	18	12.8
	Funny	15	10.6
	Hopeful	4	2.8
	Informative	46	32.6
	Nostalgic	5	3.5

## Data Availability

The raw data supporting the conclusions of this article will be made available by the authors on request.

## Appendix A

The following table (Table A1) presents the framework used to code the TikTok videos included in this content analysis.

**Table A1:** Coding framework.

Code	Definition
Presenter of content	Code the main presenter (multiple can be coded only where different presenters appear equally)
Health professionals	Identified as a health professional (or in training), for example, general practitioners, nurses, medical students, paediatricians.
Patient affected by rheumatic fever or close family member	A person who has rheumatic fever or an immediate family member of a person with rheumatic fever.
Celebrity or public figure	A celebrity, professional athlete or other well-known public figure.
Media personnel	Media personnel such as journalists, news presenters or podcasters.
Community member	A lay community member (not otherwise coded in the above categories). For example, school students, church groups, etc.
No clear presenter	In cases where there is no obvious presenter, i.e., videos of images.
Audio	Code the main audio type (multiple can be coded only where different audios are used equally)
Direct spoken language or singing	Direct speaking or singing to the camera.
Voice over	An audio narration without the image of the speaker.
Music or sounds	Only music or sounds.
No audio	No intentional audio, i.e., does not include videos where audio has been removed for copyright.
Video type [21]	Code all that are applicable
Acting or role play	A video in which information is presented via individual or group acting, and role plays, e.g., scenarios.
Animated infographics or illustrations	A video that uses a combination of images, illustrations, charts, graphs, text, cartoons and other elements that are animated to visualize information, e.g., cartoons, illustrations.
Documentary	A video that documents an experience, factual record or report of events or people, e.g., a documentary excerpt.
Oral speech or singing	A video in which speakers orally, and often formally, present information to the audience (via direct speech or singing, not voiceovers).
Pictures or pictorial slideshow	A video that presents a series of still images in a prearranged sequence.
TikTok trends and memes	A video that clearly features TikTok trends or memes, e.g., dance trends, video trends.
Primary purpose of video	Code only one
For entertainment	Videos that are primarily for entertainment, e.g., dance, activities, songs, skits.
Health advice	Videos that intend to provide health advice (typically from a health professional).
Medical education	Videos that are intended for medical education and/or study purposes.
Personal stories	Videos representing personal experiences of rheumatic fever patients and/or family members.
Rheumatic fever content	Code all that are applicable
Prevention	Mentions prevention measures for rheumatic fever such as improving housing and seeking early healthcare.
Disease pathogenesis	Mentions or describes the mechanisms by which rheumatic fever develops or progresses, such as developing from a streptococcal infection.
Symptoms	Mentions symptoms of streptococcal infections or rheumatic fever such as a sore throat, fever, unusual body movements, rashes and shortness of breath.
Testing and/or diagnosis	Identifies a testing or diagnosis regime related to streptococcal infections or rheumatic fever, e.g., sore throat swabbing, skin swabs, JONES criteria.
Medication or treatment-related	Mentions medications or treatments for streptococcal infections, rheumatic fever or rheumatic heart disease.
Outcomes of disease	Makes an explicit link from rheumatic fever to later health outcomes such as rheumatic heart disease, heart failure, premature mortality, etc.
Where to seek services	Identifies where to seek health advice or services such as health organisations, health professionals, schools, other social services, etc.
Recovery	Mentions the process or journey to “recovering” from rheumatic fever.
Misinformation	Makes reference to information that is inconsistent with current evidence or guidelines related to rheumatic fever (including both direct misinformation and cases where creators dispel misinformation).
Identifies populations that are more at risk	Mentions population groups that are more at risk of rheumatic fever such as children, adolescents, Indigenous populations, low- and middle-income countries, and those with a family history of rheumatic fever.
None apply	None of the above categories apply, e.g., videos presenting a person with rheumatic fever but not mentioning the predefined categories.
Overall connotation	Code only one
Negative/Negative leaning	Overall connotations of the video are primarily negative, for example, focused on pain, suffering.
Neutral	The general connotations are neither negative nor positive, for example, informative videos that share objective evidence about rheumatic fever.
Positive/Positive leaning	Overall connotations of the video are primarily positive, for example, videos focused on recovery, hope, being strong and resilient.
Tones [32]	Code all that are applicable
Serious	Content is presented in a solemn, sincere way.
Scary	Content is perceived to cause alarm or fear.
Sad	Content expresses sorrow, grief or unhappiness.
Lighthearted	Content is presented in a lighthearted, entertaining way.
Hopeful	Content is presented in an optimistic or inspiring way.
Funny	Content is intended to be humorous and entertaining.
Informative	Content is presented with the intent of informing, educating or providing evidence.
Nostalgic	Content is presented in a sentimental way that evokes nostalgia.
